# Prevalence of overweight and obesity based on the body mass index; a cross-sectional study in Alkharj, Saudi Arabia

**DOI:** 10.1186/s12944-018-0778-5

**Published:** 2018-06-05

**Authors:** Sameer Al-Ghamdi, Mamdouh M. Shubair, Abdulrahman Aldiab, Jamaan M. Al-Zahrani, Khaled K. Aldossari, Mowafa Househ, Shanila Nooruddin, Hira Abdul Razzak, Ashraf El-Metwally

**Affiliations:** 1 Department of Family Medicine, College of Medicine, Prince Sattam Bin Abdulaziz University, Al-Kharj, Saudi Arabia; 2 School of Health Sciences, University of Northern British Columbia (UNBC), 3333 University Way, Prince George, BC V2N 4Z9 Canada; 3 Department of Medicine, Oncology division, College of Medicine, King Saud University, Riyadh, Saudi Arabia; 4 Department of Health Informatics, College of Public Health & Health Informatics, King Saud Bin Abdulaziz University For Health Sciences, Riyadh, Saudi Arabia; 5 Aga Khan University Hospital, Karachi, Pakistan; 6 Ministry of Health and Prevention, Dubai, United Arab Emirates; 7 Docent of Epidemiology, School of Health Sciences, University of Tampere, Tampere, Finland; 8 Epidemiology & Biostatistics Department, College of Public Health & Health Informatics, King Saud Bin Abdulaziz University for Health Sciences, P. O. BOX 3660, Riyadh, Saudi Arabia

**Keywords:** Overweight, Obesity, Body-mass-index, Al Kharj; Saudi Arabia

## Abstract

**Background:**

Obesity and overweight are accompanied with several different chronic diseases. Overweight and obesity can be measured by using body mass index (BMI) and is also used widely as an index of relative adiposity among any population. The aim of the study is to evaluate the prevalence of overweight and obesity among general population in Al-Kharj, Saudi Arabia.

**Methods:**

Cross-sectional analysis was undertaken from a representative sample (*N* = 1019) of the Al Kharj population. Anthropometric measurements including the waist circumference (in centimeters), height (in meters), and weight (in kilograms) of the subjects were undertaken by means of standard apparatus. SPSS 24.0 was utilized for statistical analysis of the data.

**Results:**

Majority of respondents in this study were overweight and obese (54.3%) compared with 45.7% being non-obese. A linear positive association of increasing BMI with older age groups was present in males and females. Men had larger waist circumference, weight and height measures as compared with their female counterparts. Regression analysis showed increasing age, being married and high serum cholesterol to be the significant predictors of overweight and obesity while gender, education level, job status, and having diabetes were not.

**Conclusions:**

The obesity-overweight prevalence in the Saudi population is high mainly across both genders. However, the associated factors are potentially preventable and modifiable. The regional barriers to lifestyle modifications and interventions to encourage active lifestyles, especially among adolescents to limit the occurrence of obesity and ultimately promote health and wellbeing, are warranted. Furthermore, prospective studies are needed in future to confirm the aetiological nature of such associations.

## Background

Obesity, which broadly refers to excessive body fat, is a significant public health concern and its occurrence has reached an epidemic proportion in both developing as well as developed countries [1]. Obesity tends to impact more than one third of the population around the world. Globally, obesity is estimated to cause more than 2.8 million mortalities, 4% of YLL i.e. Years of Life Lost, and at least 35.8 million worldwide DALYs i.e. Disability-Adjusted Life Years [2]. If such secular trends continue, an estimated 20% of the worldwide population will be affected by obesity by 2030, while 38% will be overweight [3]. It is expected that 85% of U.S. citizens will be affected by obesity by 2030 [4].

The prevalence of obesity in Gulf Countries among children and adolescents ranges from 5% to 14% in males and from 3% to 18% in young females [5]. Data are scarce from other Middle Eastern countries; however, compelling evidence is present indicating the rise in obesity rates. For instance, recent surveys found that in Kuwait, 48% of females and 36% of males were obese, whereas 77% of females and 74% of males were overweight. While 44% of the female population and 28% of the male population from Saudi Arabia were found to be obese. However, 71% of women and 66% of men were reported to be overweight [6].

BMI is predisposed by certain determinants that can either be environmental or genetic. Such environmental factors include physical activity, gender, marital status, job and education level, diet, co-morbidities i.e. diabetes, hypertension, cardiac and endocrinal issues, cancers etc. [7]. In Saudi Arabia, 4% obesity was reported in rural areas. Conversely, obesity is reported to be 10% in the Western regions and 14% in the Eastern region [Jizan (12%), Riyadh (22%), and Hail (34%)] due to the consumption of more fast food and a sedentary lifestyle [8]. Regarding gender distribution, females were disproportionately affected by extreme obesity than males [6, 9–11]. Income also predicted obesity, particularly in Arab countries [12]. The role of education was clear when more obesity was found prevalent in the illiterate population as was found in Syria, Jordan and Lebanon [13, 14]. Married people were also more susceptible to being overweight and obese [9–11, 15–17].

Obesity can contribute to greater risks of non-communicable diseases (NCDs) causing morbidity and mortality. It is a modifiable and preventable risk factor for insulin resistance leading to impaired glucose tolerance [9], metabolic syndrome [18] and dyslipidemia [19]. The relative risk after adjusting the variable of age was found to be 60.9 for the development of diabetes with a BMI ≥35Kg/m^2^ [10]. Framingham study reported hypertension having the relative risks of 1.46 and 1.75 in overweight and obese young adults respectively, while the Honolulu Heart Program and Japanese survey showed its predictable effect on hypertension in the older population [20, 21].

Obesity can also contribute to ischemic stroke and Obstructive Sleep Apnea whereas 75% of adolescents with asthmatic emergencies were either obese or overweight. Gastroesophageal reflux disease, cholelithiasis, osteoarthritis, various cancers, psychiatric illnesses, polycystic ovarian syndrome in females, infertility and impotence in males were also found to be associated with obesity. Obese females were 20% less likely to get married, had lesser chance to complete school, had more poverty at the household level and less earnings in contrast to women who were not overweight [22]. Fontaine et al. concluded that a noticeable decline in life expectancy was present in obese adolescents in comparison to non-obese individuals [23]. Similarly, non-smoking obese females and males aged 40 years lived less than 7.1 and 5.8 years than their non-obese counterparts.

To assess obesity, the World Health Organization (WHO) has implemented the body mass index (BMI) scale, which can be obtained by dividing the total weight of the body in kilograms by the square of the height measured in meters, as a substitute of the over-all body fat measure [19]. With this, obesity can be well-defined if BMI value is ≥30Kg/m^2^. It usually correlates with the fraction of body fat in individuals having high prevalence of obesity [24]. As per WHO [2], an increased risk of co-morbidities exists when the BMI is between 25.0 and 29.9 while moderate-severe risk lies when it is > 30. Hence, the goal must be to sustain the range of BMI between 18.5 and 24.9 kg/m^2^. Therefore, the purpose of the study is to estimate the prevalence of overweight and obesity within the general population in Al-Kharj, Saudi Arabia.

## Methods

### Study design

A population based cross sectional study using the data drawn from the general population in Saudi Arabia between January 2016 and June 2016 was undertaken.

### Settings

The research study was conducted in Al Kharj, a city located in Al Kharj Governorate in central Saudi Arabia, 77 km south of Riyadh, with an estimated population of 376,000. It is connected to the city of Dammam and Riyadh by rail. It possesses mixed urban (military and civilian), rural, and adjacent nomadic communities.

### Sample size

Approximately 1019 (638 females and 381 males) were recruited into the study by using a multi-stage sampling method from different governmental and private institutes after obtaining verbal informed consent. The total population of these institutions were divided into groups called clusters after acquiring a list of participants in each institute nominated. Samples of the respondent were then selected using simple random sampling from each of the group (cluster).

### Selection of participants (criteria for inclusion and exclusion)

Saudi citizens, 18 years or older were selected, based on their eligibility as well as willingness to participate in the study. Non-Saudi residents, Saudi citizens younger than 18 years of age, and participants who were not willing to sign the consent form were excluded.

### Materials/instruments

A structured questionnaire was used for collecting data. The participants filled a set of questionnaires on socio-demographics including age, gender, marital status, and level of education. Anthropometric measurements body weight (in kilograms), height (in meters), body mass index (BMI), and waist circumference (in centimeters) were also included in this questionnaire.

### Consideration and procedures

In general practice, based on the standards for anthropometric measurements, the weight of an individual body was measured in socks and light clothes to the nearest 0.1 kg, using a similar digital medical scale. Height of a participant was measured to the nearest of 0.1 cm using a stadiometer precisely noted in standing position with no shoes on. Weight was measured through a digital weighing scale. Prior to the measurement, the scale was calibrated to the zero level and was also verified for repeatability of the readings. BMI was computed by the dividing weight by height in meters square (kg/m^2^) and weight categories were demarcated following the WHO standard as 30 kg/m2 obese.

Waist circumferences (WC), at the level of the hip and umbilicus circumference was measured at the widest girth of the hip using a flexible non-stretchable tape. Women with a WC of < 80 cm, 80.0 - 87.9 cm and ≥ 88 cm and males with a waist circumference of < 94 cm, 94.0 - 101.9 cm and ≥ 102 cm were termed as normal weight, overweight and obese, correspondingly.

A blood glucose monitoring system was used to measure the fasting blood glucose (FBG) of each respondent. Blood samples were obtained after a minimum of 8 h of fasting by the respondents. The results obtained with the glucometer were calibrated with laboratory outcomes using the glucose oxidase method.

Blood samples were collected from each participant by trained nurses and phlebotomy for HbA1c, fasting lipid profile (total cholesterol, triglycerides, HDL- and LDL cholesterol). A unique ID (barcode) was assigned to these patients. Two tubes were used: one for Hemoglobin A1c while the other for chemistry- then gentle rolling was applied at roller mixer for preventing clotting. Any clotted samples or critical outcomes were reported, and participants were contacted immediately for an alternative sample. The tubes were gathered in a special ice container for improved handling and care. These samples were sent to the central laboratory within 1–2 h of duration. Samples were run for the test procedures. The data obtained from all the samples/specimen were encoded in Beckman Coulter. The heparin plasma sample was used for the Chemistry analysis. The heparin vacutainers were separated from the remaining. After which, the vacutainers were organized along with the barcode numbers, and kept in the centrifuge. Samples were centrifuged for 5 min @ 4000 rpm for the separation of plasma. Once the plasma was separated, it was then utilized for the test procedure. Calibration (check and control) of the test was essential for which sample was programmed into the machine termed as Dimension Xpand Plus accordingly and results were collected after the test were finished.

### Operational definition

Overweight can be defined as a BMI of 25 and more, and obesity as an index of 30 and more [25]. DM was defined as FPG ≥126 mg/dL or self-reported history of diabetes as defined by American Diabetes Association (ADA) criteria. Pre-diabetes was defined using HbA1c cutoff level of 5.7- < 6.5%, while Diabetes Mellitus (DM) was ≥6.5%, according to the American Diabetes Association 2016.

### Data analysis

Data analysis was performed using SPSS 24.0 for Windows. Analysis involved descriptive statistics for frequencies, multivariate and logistic regression analysis. Categorical variables such as gender, educational level, and age groups were summarized and reported in terms of frequency distribution. A chi-square test was utilized to examine the association between different categorical variables whereas the t-test or ANOVA were used for continuous variables. The multinomial logistic regression model was used to examine the relationship (adjusted odds ratios) between overweight, obesity and possible contributing factors. *P*-values of <0.05 were considered statistically significant.

### Ethical approval

This was gained from the local Institutional Review Board (i.e. Committee of Scientific Research and Publication). Written permission and verbal consent was sought from the respondents before commencement of the study. Participants  were also guaranteed of the confidentiality as well as were notified that the participation would be voluntary in the study.

## Results

Table 1 illustrates the prevalence of being overweight along with obesity in males (*n* = 381) using BMI standard classification stratified by four age groups. There is a linear positive association (trend) of increasing BMI with older age groups. For example, the Class I obese (30–34.9 kg/m^2^) shows 15.4% of males in the 18–29 years; 23.3% of males in the 30–39 years; 25.5% of males in the 40–49 years; and 42.1% of males in the 50–67 years. As presented in Table 2 for females (*n* = 638) and except for the 40–49 years old, a similar linear positive trend was noted: The Class I obese females show 12.2% in the 18–29 years; 28.6% in the 30–39 years; 17.4% in the 40–49 years; and 42.9% in the 50–67 years.

**Table 1:** Prevalence of Overweight and Obesity by Age Group in Males (*n* = 381)

Age groups	Non-Obese (i.e. < 25 kg/m^2^) (*n* = 120)	Overweight (i.e. 25 to 29.9 kg/m^2^) (*n* = 123)	Class I Obese (i.e. 30 to 34.9 kg/m^2^) (*n* = 79)	Class II/III Obese (i.e. ≥ 35 kg/m^2^) (*n* = 59)
	*n* (%)	*n* (%)	*n* (%)	*n* (%)
18–29 years^a^	74 (40.7%)	53 (29.1%)	28 (15.4%)	27 (14.8%)
30–39 years^a^	31 (24.0)	48 (37.2%)	30 (23.3%)	20 (15.5%)
40–49 years^a^	14 (27.5)	17 (33.3%)	13 (25.5%)	7 (13.7%)
50–67 years^a^	1 (5.3)	5 (26.3%)	8 (42.1%)	5 (26.3%)
Total^a^	120 (31.5%)	123 (32.3%)	79 (20.7%)	59 (15.5%)

^a^Values are count (%)

**Table 2:** Overweight and Obesity by Age Group in Females (*n* = 638)

Age group	Non-Obese (< 25 kg/m^2^) (*n* = 345)	Overweight (25–29.9 kg/m^2^) (*n* = 149)	Class I Obese (30–34.9 kg/m^2^) (*n* = 90)	Class II/III Obese (≥ 35 kg/m^2^) (*n* = 53)
	*n* (%)	*n* (%)	*n* (%)	*n* (%)
18–29 years^a^	336 (61.0%)	117 (21.2%)	67 (12.2%)	31 (5.6%)
30–39 years^a^	8 (14.3)	25 (44.6%)	16 (28.6%)	7 (12.5%)
40–49 years^a^	1 (4.3)	5 (21.7%)	4 (17.4%)	13 (56.5%)
50–67 years^a^	0 (0.0%)	2 (28.6%)	3 (42.9%)	2 (28.6%)
Total^a^	345 (54.2%)	149 (23.4%)	90 (14.1%)	53 (8.3%)

^a^Values are count (%)

For the overall sample (*n* = 1019) in Table 3, it is evident that being in an older age group was significantly associated with being overweight, class I or class II/III obese. For example, the Class I obese shows 13.0% in the 18–29 years; 24.9% in the 30–39 years; 23.0% in the 40–49 years; and 42.3% in the 50–67 years (Table 3). The Class II/III obese similarly shows a significant association of BMI with increasing age: 7.9% in the 18–29 years; 14.6% in the 30–39 years; 27.0% in the 40–49 years; and 26.9% in the 50–67 years. Most of the respondents in this study are overweight and obese (54.3%) compared with 45.7% being the non-obese BMI category (< 25 kg/m^2^).

**Table 3:** Overweight and Obesity Prevalence by Age Group in the entire sample (*n* = 1019)

Age group	Non-Obese (< 25 kg/m^2^) (*n* = 465)	Overweight (25–29.9 kg/m^2^) (*n* = 272)	Class I Obese (30–34.9 kg/m^2^) (*n* = 169)	Class II/III Obese (≥ 35 kg/m^2^) (*n* = 112)
	*n* (%)	*n* (%)	*n* (%)	*n* (%)
18–29 years^a^	410 (55.9%)	170 (23.2%)	95 (13.0%)	58 (7.9%)
30–39 years^a^	39 (21.1)	73 (39.5%)	46 (24.9%)	27 (14.6%)
40–49 years^a^	15 (20.3)	22 (29.7%)	17 (23.0%)	20 (27.0%)
50–67 years^a^	1 (3.8%)	7 (26.9%)	11 (42.3%)	7 (26.9%)
Total^a^	465 (45.7%)	272 (26.7%)	169 (16.6%)	112 (11.0%)

^a^Values are count (%)

Table 4 demonstrates weight (in ‘kg’), height (in ‘m’) and waist circumference (in ‘cm’) for men, women, and the overall sample. Men tended to have larger WC more than females (mean WC = 96.76 and 75.54 respectively). Weight and height measures were also higher in males compared with their female counterparts (Table 4).

**Table 4:** Body weight, height, and WC measures for the study population (*n* = 1019)

Variables	Men (*n* = 381)	Women (*n* = 638)	Total (*n* = 1019)
Mean (SD)			
Weight (kilograms)	83.83 (21.1)	63.08 (16.04)	70.84 (20.71)
Height (meters)	1.71 (0.08)	1.57 (0.06)	1.62 (0.09)
Waist circumference (centimeters)	96.76 (23.30)	75.54 (14.62)	83.47 (21.02)

When diabetic status was examined according to BMI categories (Table 5), we found that the proportion of diabetic individuals was positively and significantly associated with being in a higher BMI category: 11.4% in the non-obese; 22.7% in the overweight; 38.6% in the Class I obese; and 27.3% in the Class II/III obese (Table 5). This observation is consistent with previous research that indicated increasing risk of diabetes with increasing body weight status (BMI).

**Table 5:** Prevalence of Overweight and Obesity by Diabetic Class (*n* = 1019)

Diabetic class	Non-Obese (< 25 kg/m^2^) (*n* = 465)	Overweight (25–29.9 kg/m^2^) (*n* = 272)	Class I Obese (30–34.9 kg/m^2^) (*n* = 169)	Class II/III Obese (≥ 35 kg/m^2^) (*n* = 112)
	*n* (%)	*n* (%)	*n* (%)	*n* (%)
Diabetic^a^	5 (11.4%)	10 (22.7%)	17 (38.6%)	12 (27.3%)
Pre-diabetic^a^	65 (28.1%)	66 (28.6%)	54 (23.4%)	46 (19.9%)
Non-diabetic^a^	395 (53.2%)	196 (26.4%)	98 (13.2%)	54 (7.3%)
Total	465 (45.7%)	272 (26.7%)	169 (16.6%)	112 (11.0%)

^a^Values are count (%)

We further examined diabetic status for each of the four age groups (Table 6). The results showed that the prevalence of diabetes was linearly and positively associated with age. The Cochran-Armitage trend test available from the contingency table of 4 age groups-by- 3 diabetic status groups in SPSS 24.0 for Windows was significant (Linear-by-Linear association = 214.070, *P* < 0.0001). There was a significantly positive linear association with having diabetes in older age groups: 13.3% in the 18–29 years were diabetic; 26.7% in the 30–39 years were diabetic; 33.3% in the 40–49 years were diabetic; and finally, 26.7% in the older age group (50–67 years) were diabetic (Table 6).

**Table 6:** Prevalence of Diabetic Status by Age group (*n* = 1019)

Age group	Diabetic (*n* = 45)	Pre-Diabetic (*n* = 231)	Non-Diabetic (*n* = 743)
	*n* (%)	*n* (%)	*n* (%)
18–29 years^a^	6 (13.3%)^b^	117 (50.6%)	610 (82.1%)
30–39 years^a^	12 (26.7%)^b^	72 (31.2%)	102 (13.7%)
40–49 years^a^	15 (33.3%)^b^	35 (15.2%)	24 (3.2%)
50–67 years^a^	12 (26.7%)^b^	7 (3.0%)	7 (0.9%)
Total	45 (100%)	231 (100%)	743 (100%)

^a^Values are count (%)

^b^Linear-by-Linear Association = 214.070, *P* < 0.0001

### Multinomial logistic regression analysis

We conducted a multinomial logistic regression analysis by regressing the categorical BMI outcome variable on sociodemographic variables (age; gender; marital status; education level; and job status) and other variables which included blood cholesterol and whether a subject has diabetes or not (Table 7). The non-obese BMI category (< 25 kg/m^2^) was used as the reference category to test the odds ratio (Exp^^^B) for each of the other three BMI categories: the overweight; class I obese; and class II/III obese.

**Table 7:** Multinomial logistic regression showing significant predictors of each BMI class using odds ratio and corresponding 95% confidence interval^a^

Overweight				
Variable	Odds Ratio (Exp (B))	*P*-value	95% CI	
			Lower	Upper bound
Intercept	–	–	–	–
Cholesterol	1.419	.001	1.153	1.746
Single/unmarried	1.367	.000	1.236	1.570
Job status (civilian worker)	1.607	.049	1.002	2.577
Class I obese				
Intercept	–	–	–	–
Age	1.045	.020	1.007	1.084
Cholesterol	1.606	.000	1.269	2.034
Single/unmarried	1.542	.021	1.323	1.911
Job status (civilian worker)	1.303	.035	1.100	1.919
Class II/III obese				
Intercept	–	–	–	–
Age	1.050	.015	1.010	1.093
Cholesterol	1.575	.001	1.207	2.056
Job status (civilian worker)	2.018	.042	1.025	3.976

^a^The reference category is the non-obese (< 25 kg/m^2^)

The relative risk of being overweight was associated with higher cholesterol (OR = 1.419, *P* = 0.001), being single/unmarried (OR = 1.367, *P* < 0.0001), and being a civilian worker (OR = 1.607, *P* = 0.049). Being classified as Class I obese was associated with statistically significant risk of older age (OR = 1.045, *P* = 0.020), higher cholesterol (OR = 1.606, P < 0.0001), being unmarried (OR = 1.542, *P* = 0.021), and having diabetes (OR = 1.303, *P* = 0.035). Whereas being classified as Class II/III obese was significantly associated with older age (OR = 1.050, *P* = 0.015), higher cholesterol (OR = 1.575, P = 0.001), and being a civilian worker (OR = 2.018, *P* = 0.042).

The adjusted R-squared (R^2^) for the multinomial logistic regression model is represented by the Cox and Snell Pseudo R-Square in the SPSS output for this model, with a value of 0.194.

## Discussion

Our study findings show that the vast number of the study population was overweight and obese. Its prevalence across both genders showed a linear and positive trend with older age groups with an exception of the 40–49 female age group. Hence, making it evident that increasing age was significantly associated with overweight, class I or II/III obese. While evaluating the measures of obesity/overweight, males were found to have greater values of WC, height and weight in comparison to their female counterparts. It was also noted that the proportion of diabetic individuals also had significant positive association with being in a higher BMI category. Significant predictors of high BMI also included high serum cholesterol, being married and increased age during regression analysis while gender, diabetes, job and educational status were not significant.

A major strength of our study was the usage of cluster designs for surveys as recommended by the WHO which assures that the nominated sample specifies the entire population precisely. We included common risk factors and evaluated their association with a high BMI. Moreover, trained nurses collected the data as well as anthropometric measurements in our study to ensure the reliability of the data and the anthropometric and clinical measurements. Our study has used the more recent standard globally accepted WHO/NHLBI criteria for the definition of BMI cut-off values that would be applicable to all countries/regions including the Gulf region.

Literature shows that high BMI can be the root cause for many of non-communicable diseases (NCDs) in the world. The majority of respondents in this study were also overweight and obese (54.3%), which has become an increasing trend around the world with increasing prevalence rates in the USA (36.5%) [3], Spain (29%) [16], Greece (23%) [15], Lebanon (17%) [26], Kuwait (43%), Saudi Arabia (35%) and Qatar (33%) [12]. A study conducted in the city of Jeddah, Saudi Arabia, found that 33.8% of women were found to be obese and 47% with WC > 80 cm [27] whereas in our study the mean WC in females was 75.54 cm. On the other hand, there remains a perception in parents that overweight children are a sign of prosperity, beauty, fertility, prosperity and high social status [28].

Obesity was also found to be associated with certain factors such as diabetes in our study where its contribution towards diabetes was also supported by other international and national published work in India [29], USA [9, 10], China [30], Lebanon [26] and Saudi Arabia [31]. Regarding gender differences, our study suggested that males tend to have more height, weight and WC than females. Studies from China [1], Norway [4], USA [3] and Greece [15] also revealed that the incidence of obesity was more in men as compared to women. However, women were also found to have more obesity than men in the USA [31], Finland [32], Canada [33], Lebanon [26] and Saudi Arabia [5].

A linear and positive trend was obtained between increasing age and high BMI across both genders in our study. Likewise, large population studies indicate that BMI gradually increases during adult life reaching a peak at 50–59 years in males as well as in females, showing a declining BMI trend after the age of 60 years [31, 34–36]. Consistently, in Lebanon, men and women had a linear association between increasing age and obesity in 20–60 years and 20–70 years respectively [26]. In Spain, high BMI figures amplified continuously from 10% as identified in the age cluster of 18 to 25 years to more than 50% in above 55 years of age [16].

Being married was a significant predictor of high BMI in our study. The companionship after marriage may encourage an individual to avoid obesity or even contribute towards it. In the same context, in Greek [15], Turkish [17] and Spanish [16] countries, marital status was found significantly related to obesity. As per Kilicarslan and colleagues [17], risk of being obese was 2.5 times greater in individuals who were married as compared to those who were single, divorced or widowed. Similarly, in Iran, threefold higher risk was found for married men and women [18]. In Americans, married males were 21% more prone to become overweight, whereas wedded females had a 21% decreased probability of being obese. However, a study conducted in Japan showed no relationship between body weight and marriage [11]. On the contrary, this relationship was not evident in Malaysians and Americans [36].

Klop et al., [18] reported  a linear connection between the grade of obesity and serum cholesterol levels which was in conjunction with our study. They observed higher concentrations of mean total cholesterol and triglycerides whereas low level of High Density lipoproteins (HDL) in obese persons when compared to normal weight subjects [37]. An imperative link amongst dyslipidemia and obesity seems to be the development of insulin resistance [18]. This mechanism along with Obesity, hypo HDL cholesterolemia, hypertriglyceridemia and glucose intolerance are characteristics of insulin resistance disorder which is also extensively widespread among the Saudi inhabitants of age 40 years and above [22] in addition to Australians [38] and residents of the Pacific Islands.

Educational level was not found as a significant predictor of obesity in our study. In contrast to Greek individuals where the hazard for being obese was lesser in educated females in comparison to illiterates with no significant differences among males. As per the bi-ethnic study [11], education level was found to be a significant and vital predictor of high weight for Americans but not for Japanese. As in the USA, with each year of education, the possibility of being overweight or obese was reduced by 2–9% [9]. Likewise, in Turkey, 62% university students had normal weight while 31% were obese [17], while, in Lebanon, less education was associated with high BMI [26].

Our study could not find significant association between job status and high BMI. Whereas, a previous study from the same region demonstrated an increased BMI associated with increased monthly income [34]. In a longitudinal study, Seiluri [22] found blue-collar workers to have 50% more chances of being insufficiently active in comparison to professional and white-collar workers. Contrary to these findings, obesity was found associated with unemployment in Turkey [17]. Hence, such remarkable finding gives us a message to initiate preventive measures and health education to prevent the Saudi population from the vast health effects and later complications of obesity.

Finucane, Stevens, and Cowan [39] assessed a worldwide escalation in average BMI of 0.4 kg/m^2^ per decade while The HUNT study records [5] showed it to be 1.0 kg/m^2^ in comparison to 1.1 kg/m^2^ for America. An augmented level of all-purpose health education provided at population level might contribute to its decrease as its consequences can be harmful for a nation. For instances, it is reported that with every 1 kg increase  in weight, the occurrence of diabetes escalates up to 9% [35] thus, it is extremely important to hamper this conversion at early levels to prevent our population from many other obesity-related diseases.

There are a few limitations in this study. Primarily, the cross-sectional design of the study limited the causal inference. The targeted age groups were consistent with the previous published literature yet both extremes could have been enhanced to see the risk factors in young as well as in an old age population. Additionally, various other socio-demographic characteristics, marital status, family history, diet, physical activity and concomitant illnesses should also be considered while considering risk factors for being overweight and obese.

## Conclusion

Obesity is preventable and understanding its prevalence, associated factors across different geographic regions and socio-demographics is the key to our efforts in designing culturally suitable and relevant health promotion activities. Obesity in adulthood can be taken as a powerful predictor for mortality in older ages. An alarming global situation of increasing trends of obesity is associated with large decreases in life expectancy. Additionally, important risk factors like being married; increasing age, high cholesterol etc. were also highlighted which was in line with previous literature. Analysis in the study showed that BMI significantly increased with age and this might elevate the risk of NCDs in the next adult generation and have a noteworthy influence on the financing and provision of future health-care services in Saudi Arabia. Therefore, in the light of such findings it is now particularly vital to speed up health-promotion behaviors to construct effective interventions.

Well-designed prospective studies are needed in the future to study the etiological nature of this relationship. Further studies are also required to make causal inferences and to examine certain barriers to physical activity and economic, social, cultural and behavioral factors leading to high BMI in the Saudi population.

## Abbreviations

BMI: Body Mass Index
DALY: Disability-Adjusted Life Years
NCDs: Non-Communicable Diseases
WC: Waist Circumference
WHO: World Health Organization
YLL: Years of Life Lost
